# Adverse Effects Associated with Dermal Filler Treatments: Part II Vascular Complication

**DOI:** 10.3390/diagnostics14141555

**Published:** 2024-07-18

**Authors:** Gi-Woong Hong, Hyewon Hu, Kathleen Chang, Youngjin Park, Kar Wai Alvin Lee, Lisa Kwin Wah Chan, Kyu-Ho Yi

**Affiliations:** 1 Samskin Plastic Surgery Clinic, Seoul 06577, Republic of Korea; cosmetic21@hanmail.net; 2 Division in Anatomy and Developmental Biology, Department of Oral Biology, Human Identification Research Institute, BK21 FOUR Project, Yonsei University College of Dentistry, 50-1 Yonsei-ro, Seodaemun-gu, Seoul 03722, Republic of Korea; wonhuh@yuhs.ac; 3 Harmony Aesthetic Clinic, Adelaide, SA 5000, Australia; harmonyaesthetica@gmail.com; 4 Obliv Clinic, Incheon 21998, Republic of Korea; youngjinp@gmail.com; 5 EverKeen Medical Centre, Hong Kong; alvin429@yahoo.com (K.W.A.L.); drchan.everkeen@gmail.com (L.K.W.C.); 6 Maylin Clinic (Apgujeong), Seoul 06001, Republic of Korea

**Keywords:** vascular complications, dermal fillers, ischemia, tissue necrosis, hyaluronic acid, blindness, pulmonary embolism, injection techniques, cannulas, hyperbaric oxygen therapy

## Abstract

Vascular complications arising from dermal filler treatments pose significant risks, including ischemia, tissue necrosis, and severe outcomes like blindness and pulmonary embolism. This study investigates the mechanisms of vascular complications, categorizing them into extravascular compression and intravascular emboli. Extravascular compression occurs when injected fillers compress adjacent blood vessels, leading to ischemia and potential necrosis, while intravascular emboli result from fillers entering blood vessels, causing blockages. The study emphasizes the importance of anatomical knowledge, careful injection techniques, and early intervention. Management strategies include the use of hyaluronidase to dissolve HA fillers, vasodilators to improve blood circulation, and hyperbaric oxygen therapy. The regions most susceptible to complications align with major arterial pathways, particularly the nasolabial folds and nasal region. The study also highlights the need for meticulous injection techniques, the use of cannulas over needles in high-risk areas, and the aspiration test to detect vessel penetration. Early detection and immediate intervention are crucial to mitigate adverse outcomes. Continuous education and training for practitioners, along with advancements in filler materials and injection methods, are essential for improving the safety of cosmetic procedures. This comprehensive understanding aids in preventing and managing vascular complications, ensuring better patient outcomes. The field of dermal filler treatments is advancing with new techniques and technologies, such as High-Resolution Ultrasound, Infrared Imaging, self-crossing hyaluronic acid filler, biodegradable microspheres, and microinjection.

## 1. Introduction

Vascular complications associated with filler injections can be primarily attributed to two mechanisms. The first involves damage to a blood vessel, which can result in bleeding and swelling. Filler particles entering a vessel may incite an inflammatory response, obstructing the blood supply [1,2]. Additionally, the hydrophilic nature of the injected filler may cause surrounding tissues to swell, creating extravascular compression that hampers blood circulation and potentially leads to localized necrosis of skin or tissue [3,4]. In continuation of Part 1 of our review on the adverse effects of dermal fillers, we now discuss vascular complications.

Prompt and accurate diagnosis followed by immediate intervention is essential to mitigate the adverse effects of vascular complications. Distinguishing symptoms include bruising or ecchymosis, idiopathic edema (angioedema), inflammation, and the Tyndall effect (Rayleigh scattering) [5,6].

Vascular complications may manifest differently depending on whether they occur in arteries or veins. Arterial complications can cause skin and tissue necrosis, blindness, and cerebral infarction, whereas venous issues may lead to necrosis and pulmonary embolism (Table 1) [7].

## 2. Extravascular Compression

Extravascular compression occurs when a lump of injected filler compresses an adjacent artery, potentially leading to ischemia and subsequent necrosis in the tissue supplied by that artery. Venous compression can cause congestion, impairing the flow in adjacent arterial vessels, diminishing oxygen delivery, and potentially resulting in the slow progression of necrosis [1,8].

## 3. Intravascular Emboli

Intravascular emboli, unintentionally formed inside a vessel due to filler injections, can obstruct the vessel, cutting off the oxygen supply to the tissue, and leading to potentially fatal complications [9].

Blockage of the central retinal artery or its branches may result in blindness or eye movement impairments, while arterial blockages leading to the brain could cause cerebral infarction [5,6,7].

Furthermore, filler injected into a vein may follow the venous drainage pathway, potentially resulting in a pulmonary embolism, illustrating the critical need for vigilance in injection techniques and monitoring for any signs of vascular complications immediately following the procedure [5,10].

## 4. Primary Sites of Occurrence

One of the most severe vascular complications observed is blindness, which has been reported following the inadvertent injection of filler material into arteries such as the supratrochlear, supraorbital, and dorsal nasal. The injection pressure can cause the fillers to travel retrograde within these vessels, potentially occluding the central retinal artery that supplies the optic nerve and eye, leading to blindness. If the filler material migrates into any vessel linked to these arteries and blocks the central retinal artery, there is a heightened risk of blindness [11,12].

Pulmonary embolism, though less commonly associated with fillers than with fat grafting, is most likely to occur when fillers are administered into the sentinel vein or the middle temporal vein in the temple area. Given the intricate network of facial vessels, caution must be exercised as complications such as skin and tissue necrosis, blindness, and pulmonary embolism can arise regardless of the treated area [5,13].

## 5. Skin and Tissue Necrosis

Skin and tissue necrosis, arising from vascular complications related to filler injections, is a relatively common clinical occurrence that necessitates a differential diagnosis from other general complications. The stages of necrosis, the associated symptoms, and the foundational treatment principles are critical to understand for effective management. These include:

Initial Signs: Early indicators might include discoloration and mild pain at the injection site, suggesting minor vascular impairment.

Progressive Symptoms: Without timely intervention, the area may exhibit increased discoloration, significant swelling, and intensified pain, potentially leading to ischemia and tissue death.

Advanced Necrosis: At this stage, extensive tissue death occurs, characterized by blackened skin areas and severe pain, with a risk of systemic complications if infection occurs.

Management Principles: Immediate and appropriate therapeutic interventions are crucial, ranging from pharmacological treatments to improve blood circulation and reduce inflammation, to surgical excision of necrotic tissue in severe cases.

Recognizing and addressing these stages promptly can prevent permanent damage and ensure patient safety in the administration of facial fillers (Table 2) [14].

## 6. Causative Factors of Necrosis in Filler Injections

Necrosis following filler injections can often be attributed to several causative factors related to the characteristics of the injection site and procedural techniques. Regions of the face with thicker skin are particularly prone to necrosis due to the likelihood of extravascular compression. In these areas, the dense skin reduces the available space between the skin and periosteum, leading to a higher probability of compressing the vessels within these confined spaces. For instance, the nasal tip, characterized by its thick skin and tightly adherent subcutaneous tissues, offers minimal space between the skin and underlying cartilage. Consequently, even minimal amounts of filler in such a tightly bound area can compress the arteries, significantly elevating the risk of inducing skin and tissue necrosis [15]. The situation is exacerbated if the arteries in these regions lack adequate anastomoses with adjacent vessels, increasing the susceptibility to necrosis from compressive forces exerted by the filler.

Procedural factors also play a critical role, where excessive injection of filler into a single area may compress underlying vessels, particularly in the subcutaneous fat layer, which is richer in vascular structures compared to the periosteal layer and thus more vulnerable to compression and damage.

Additionally, patients with a history of surgeries, fat grafting, other filler treatments, or the use of energy-based devices like lifting lasers may experience fibrotic changes in tissues, diminishing their flexibility and vascular resilience [2,16]. This condition predisposes these areas to higher risks of vascular damage and extravascular compression when overfilled.

In cases of necrosis driven by intravascular occlusion, notable factors include the high injection pressure that may force the filler to flow retrograde into a vessel and the volume of filler sufficient to block the vessel. To mitigate these risks, it is imperative to administer fillers with gentle and slow pressure, ensuring that the filler is injected in small, controlled amounts across multiple sessions to achieve a harmonious enhancement without overwhelming the vascular structures [1].

## 7. Prognostic Factors

The scope and severity of skin and tissue necrosis resulting from intravascular embolism are substantially more extensive than those caused by extravascular compression, which profoundly influences the prognosis. Complete occlusion of the main arteries due to embolism results in a broader and more severe range of skin necrosis compared to occlusions in peripheral arteries. The diameter of the occluded vessel is critical in determining the prognosis for skin and tissue necrosis. Recovery potential after necrosis, whether from intravascular emboli or extravascular compression, depends on the vascular anastomosis with adjacent vessels. If collateral blood flow from adjacent vessels is present, some level of tissue recovery is achievable. Conversely, in the absence of such vessels, the necrosis can be irreversible and lead to more severe complications [17,18].

## 8. Stages and Symptoms

The manifestation of symptoms in skin and tissue necrosis varies according to the stage at which it is detected and the efficacy of the initial response. Accurate identification of symptoms at each stage and the provision of appropriate treatment are crucial. The stages and their corresponding symptoms include:

### 8.1. Impending Necrosis State

**Figure 1:**
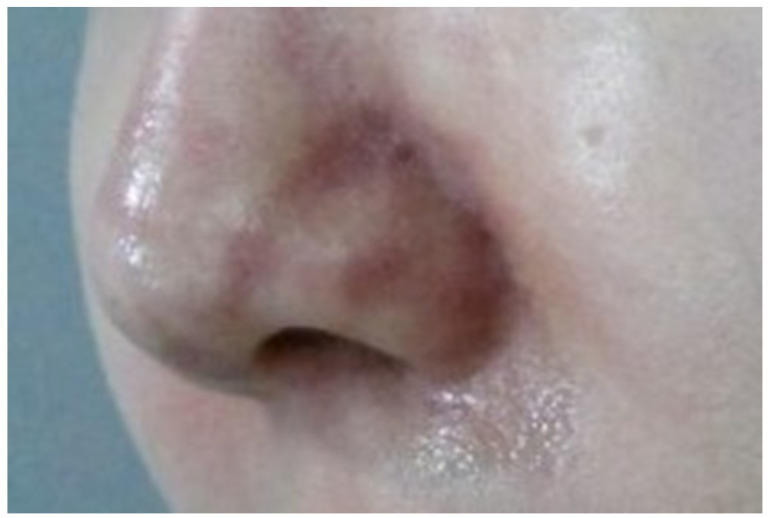
Ischemic state with purple skin of reticular pattern.

This initial stage, often termed the ischemic state, precedes the appearance of pustules on the skin. Symptoms such as a reticular pattern of purple skin discoloration along with pain and swelling typically develop gradually over 2–3 days post-procedure (Figure 1). In mild cases, this state may not progress further and can resolve without lasting effects. However, progression can lead to infection and mild eschar formation. In severe cases, where complete occlusion occurs, symptoms may manifest immediately or within hours post-procedure, rapidly advancing to more serious infections and extensive eschar formation. Prompt and intensive treatment is required if significant discoloration is noted soon after the procedure [9].

### 8.2. Infection and Wound Healing State

**Figure 2:**
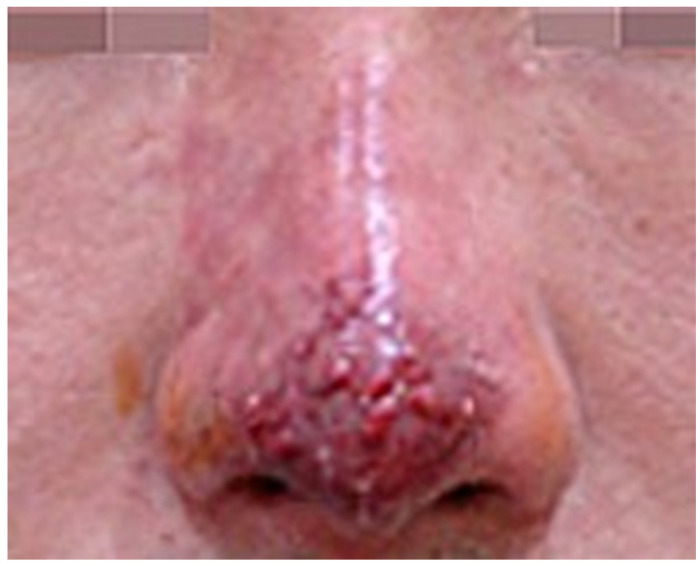
Infection state with pustule formation.

**Figure 3:**
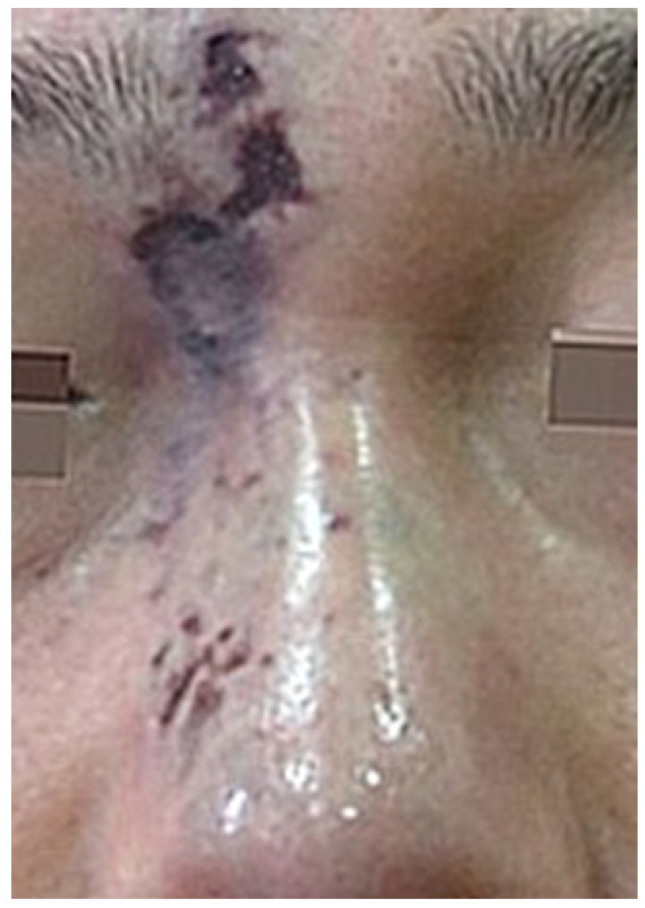
Wound healing state with eschar formation.

If the impending necrosis persists, the skin barrier deteriorates, leading to bacterial infection characterized by painful, swollen pustules. As the wound healing process advances, these pustules may rupture, forming an eschar (Figure 2 and Figure 3) [19,20].

### 8.3. Skin Defect and Scar Formation State

Following the resolution of the infection and the disappearance of the large eschar, a visible skin defect may emerge, accompanied by erythematous scarring, marking the final stages of the necrotic process [21].

### 8.4. Decompression

The degree and severity of necrosis can vary widely. Extensive necrosis typically shows symptoms within 1–2 days, whereas smaller affected areas may take longer to manifest visible signs. Ischemic symptoms arise when blood vessels are compressed by the mass of the filler or associated swelling, making decompression a critical step in alleviating the source of vascular compression and mitigating further damage. This comprehensive understanding aids in managing the progression and treatment of skin and tissue necrosis resulting from vascular complications associated with filler injections [13,22].

Initial manifestations of necrosis, such as altered skin color following a procedure, necessitate swift intervention. One therapeutic approach involves massaging the area to disperse the injected filler, thereby decreasing local pressure and enhancing blood circulation. In cases involving hyaluronic acid (HA) fillers, the administration of hyaluronidase prior to massage can facilitate the breakdown of the filler, lessening the compressive effects on surrounding tissues [2,17].

### 8.5. Hyaluronidase

Hydrolase, specifically hyaluronidase, is effective for degrading HA fillers, thus mitigating the compressive forces exerted by the filler. Notably, hyaluronidase is a polymer and may not effectively permeate the filler mass without simultaneous massage. Massaging the area spreads the filler, increasing the surface area in contact with hyaluronidase and boosting its efficacy. Research indicates that hyaluronidase can traverse blood vessel walls and dissolve intravascular HA, thus aggressive application in the vicinity of affected vessels is advocated to possibly reduce vascular occlusion [9,12,14,17,18,23,24,25,26,27,28,29,30,31,32,33,34,35,36].

### 8.6. Puncture and Drainage

For non-HA fillers, which are devoid of a specific dissolving agent, physical decompression techniques are essential. Direct removal of superficial fillers through incision and manual extraction is recommended during the initial stages of detected necrosis. This approach is discouraged if eschars have begun to form over several days, as it could exacerbate damage to already compromised skin and tissues. In instances of deeper filler placements, below the superficial musculoaponeurotic system (SMAS) layer, saline injection to dilute the filler followed by aspiration with a large bore needle may be employed [37,38].

### 8.7. Warm Massage

Where vascular compression is suspected due to densely packed filler, a press-and-roll massage technique may be beneficial. Employing warm saline-soaked gauze during the massage can aid in dilating blood vessels, facilitating decompression. Caution is imperative during physical massage to avoid exacerbating damage to fragile vascular structures, particularly in scenarios involving severe skin infections or the presence of eschar [39,40].

### 8.8. Revascularization

Following the initial decompression interventions for impending necrosis, it is crucial to implement therapeutic measures aimed at enhancing blood circulation within the necrotic regions. Vasodilators, such as Prostaglandin E1 (PGE1), are extensively utilized for the treatment of pressure ulcers and necrotic tissues. These can be administered intravenously to alleviate skin necrosis subsequent to filler injections. The standard treatment involves the gradual infusion of PGE1, diluted in 500 cc of saline, administered over a period exceeding two hours. This slow infusion rate is designed to prevent the onset of headaches that can result from rapid vasodilation. Typically, this treatment extends over three to five days, continuing until a noticeable clinical improvement in blood flow is achieved [41,42].

Additionally, aspirin is employed to decrease blood viscosity, thereby enhancing the flow through constricted vessels. This action is particularly effective in preventing or decelerating the progression of ischemic symptoms that arise from vascular occlusions related to filler applications.

Hyperbaric oxygen therapy (O_2_) is also increasingly recognized for its efficacy in boosting overall blood circulation, thus supporting the recovery process in affected areas [43,44].

## 9. Infection and Wound Healing State Treatment

### 9.1. Dressing and Infection Management

Post-procedure skin discoloration without additional complications can often be managed effectively with oral antibiotics. However, if pustules develop in conjunction with the discoloration, sterile removal and treatment become imperative. Under normal circumstances, allowing the infected area to dry might result in the formation of eschars, potentially leading to scarring. Therefore, subsequent to the removal of pustules, it is essential to apply an antibiotic gauze and maintain a moist dressing environment. This approach helps minimize eschar formation and facilitates the healing process, preventing further deterioration and enhancing recovery outcomes [45,46].

### 9.2. Wound Care

In the management of necrotic skin, the application or injection of growth factors plays a crucial role in promoting wound healing. Agents commonly utilized include Epidermal Growth Factor (EGF), Polydeoxyribonucleotide (PDRN), Platelet-Rich Plasma (PRP), and stem cells. When administering these substances, precautions must be taken to prevent bleeding. In situations where eschars are present or direct injection into lesions is challenging, peripheral injections around the wound site can be advantageous [7,40,47].

### 9.3. Treatment for Skin Defect and Scar Formation State

Effective management post skin necrosis is vital for reducing eschar formation and minimizing scarring. Untreated or severely infected wounds may lead to extensive and permanent scarring. Following the resolution of an eschar, any remaining skin depression indicates ongoing healing. As previously discussed, the use of growth factors can significantly enhance skin regeneration. For established scars, therapeutic options such as vascular or fractional lasers have been shown to effectively reduce scar visibility. In more severe cases, surgical interventions, such as skin grafting, may become necessary [38,48].

### 9.4. Important Considerations for Early Detection

It is imperative for clinical staff to proactively engage with patients who have undergone filler injections, particularly the day after the procedure, to monitor their condition. Symptoms indicative of vascular occlusion generally manifest on the day of the procedure or shortly thereafter. In instances of partial, non-severe occlusion, patients may only experience mild pain and swelling without significant skin discoloration. Differentiating these symptoms from those of skin infections is crucial, and monitoring for changes in skin color or pattern within 2–3 days post-procedure is recommended [14].

Should there be reports of discoloration or discomfort during post-procedure phone assessments, patients should be advised to send photographs of the affected area and to visit the clinic for a direct examination. Whenever possible, it is preferable for the attending practitioner to personally evaluate the treatment area.

Clinics that primarily administer HA filler treatments are advised to equip themselves with an emergency kit containing hyaluronidase and vasodilators to promptly address any incidences of skin and tissue necrosis (the use of hyaluronidase in aesthetic practice). For clinics with limited experience in handling necrosis or those without an emergency kit, establishing a network of experienced nearby physicians for immediate consultation or patient referral is crucial. Early detection and timely intervention are essential for effectively managing necrosis and preventing further complications [42].

For healthcare providers with less experience, recognizing the subtle signs of necrosis that manifest 1–2 days post-procedure is critical, as these signs may often be mistaken for minor issues such as bruising or simple infections. Educating oneself on the distinctive symptoms of necrosis and maintaining vigilant observation of the patient’s treatment area are key strategies to minimize complications arising from skin and tissue necrosis [10,14].

## 10. Blindness from Filler Injection

Blindness is a profound and devastating complication of filler injections, typically resulting from arterial occlusion. This severe outcome is predominantly due to the obstruction of the central retinal artery, which is essential for supplying blood to the optic nerve and retina. Such occlusions can occur from both synthetic fillers and autologous fat transfers [11,49,50].

### 10.1. Mechanism

**Figure 4:**
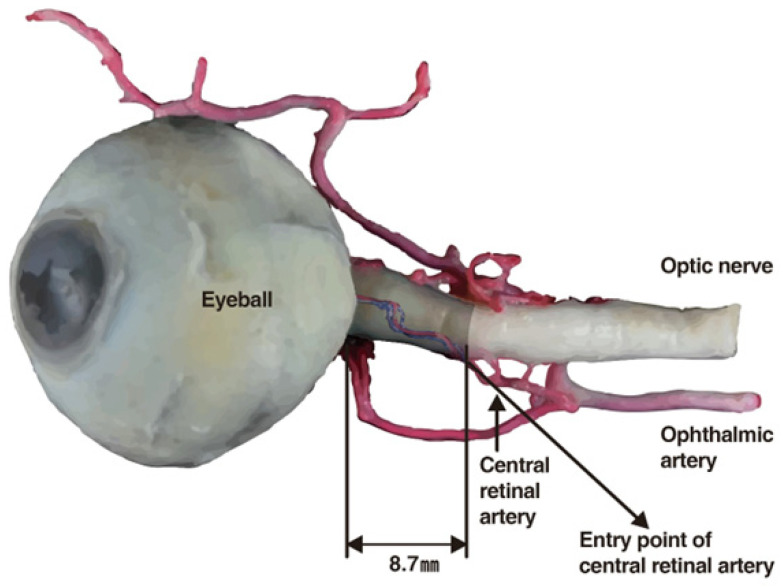
Entry point where central retinal artery goes through the optic nerve.

**Figure 5:**
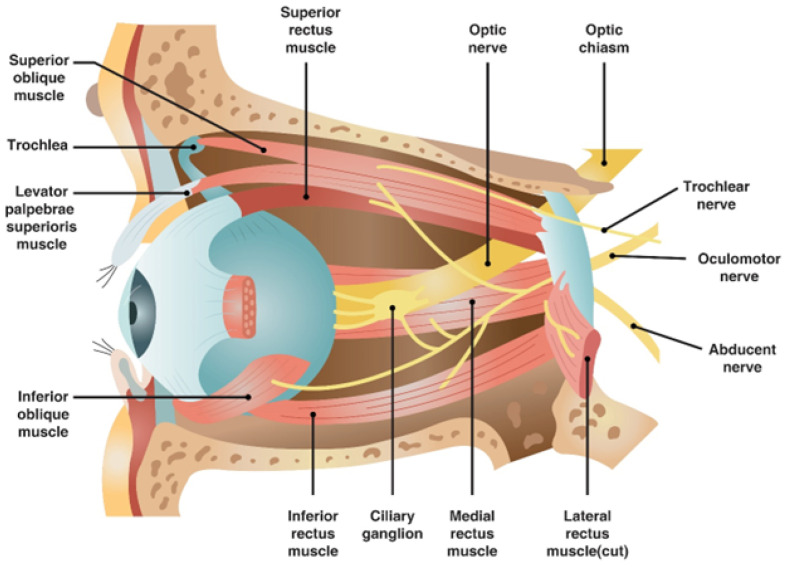
The pathway of the optic nerve and relationship with other anatomical structures. The rights for the figure belong to the authors.

The central retinal artery originates from the ophthalmic artery itself, a branch of the internal carotid artery (Figure 4). Should filler or fat particles enter and obstruct this artery, the resultant necrosis of the optic nerve leads to irreversible vision loss (Figure 5). Typically, fillers do not directly enter the central retinal artery during procedures; instead, they are inadvertently forced into facial arteries and may travel retrograde, thereby reaching and blocking the central retinal artery or, on rarer occasions, the posterior ciliary arteries, which also serve the eye [11,49,50].

The likelihood of such occurrences increases with injections in proximity to areas vascularized by branches of the ophthalmic artery, including the supratrochlear, supraorbital, and dorsal nasal regions. Nonetheless, due to extensive vascular interconnections, injections in any facial zone could potentially prompt retrograde migration of filler material into the ophthalmic artery under high injection pressures [12,51].

### 10.2. Common Sites of Occurrence

Areas such as the eyebrows, forehead, and nasal regions are particularly susceptible to complications given their dense vascularization by branches of the ophthalmic artery. Additionally, anatomical connections between the internal and external carotid systems mean that injections into other facial regions, such as the nasolabial folds, cheeks, or lips, might also allow the retrograde flow of filler into the ophthalmic artery and its branches [8,33].

### 10.3. Emergency Treatment

Once blindness ensues from vascular filler injection, physical removal of the blockage is nearly impossible. However, in scenarios involving hyaluronic acid (HA) fillers, the enzyme hyaluronidase can be employed to dissolve the filler and potentially relieve the obstruction [12,17,26].

Use of Hyaluronidase: This enzyme is capable of breaking down HA fillers that may have inadvertently entered the central retinal artery, possibly restoring blood flow. Its effectiveness in dissolving emboli within the artery has been supported by animal studies. Immediate administration following the onset of blindness symptoms is essential and is typically performed via a retrobulbar injection [23,51].

Retrobulbar Injection: To administer hyaluronidase effectively, it is injected into the retrobulbar space, allowing it to permeate the vascular system and dissolve the occluding filler. A thorough understanding of the orbital anatomy is crucial for performing this injection safely.

In summary, while hyaluronidase can significantly reduce the damage inflicted by HA fillers, its rapid and precise application is crucial. Individuals experiencing sudden vision loss following filler injections should immediately seek ophthalmological evaluation and treatment, alongside urgent measures to dissolve the filler. Prompt and decisive action is key to potentially salvaging vision and averting permanent blindness [12,51].

### 10.4. Prevention of Blindness

To avert the inadvertent arterial injection of fillers, employing meticulous techniques is crucial:

Proper Injection Depth: Administering fillers in regions with reduced vascular density, such as the supraperiosteal plane, substantially diminishes the risk of arterial penetration.

Use of Cannulas Over Needles: Although cannulas are blunter than needles, which generally lowers the risk of arterial injury, they do not entirely eliminate the risk. There have been reports of visual impairments associated with the use of cannulas, even with larger sizes, during filler injections and fat grafting (Table 3) [13,40,52].

## 11. Pulmonary Embolism

Pulmonary embolism represents a grave complication potentially arising from fillers, particularly noted with autologous fat transfers into facial regions, and can be fatal. Instances of pulmonary embolism have also emerged from filler applications intended for perineal rejuvenation [6,7].

### 11.1. Mechanism

The propensity for pulmonary embolisms increases in regions harboring large veins, such as the temporal area, where substantial veins like the middle temporal v. and sentinel v. are present. Filler entering these veins may migrate through venous channels to the pulmonary arteries, creating blockages (rare and serious events following botulinum toxin and soft tissue filler administration).

### 11.2. Prevention

Effective strategies to prevent pulmonary embolism from temporal filler injections include:

Understanding Venous Anatomy: Profound knowledge of the course and depth of veins such as the sentinel v. and middle temporal v. is essential. The sentinel v. often traverses superficially, crossing the subcutaneous layer and penetrating the superficial temporal fascia (STF) and deep temporal fascia (DTF).

### 11.3. Injection Techniques

Compartment Injection: It is advisable to use cannulas for injecting medium viscosity fillers into the compartment between the STF and DTF, under the SMAS layer, for both safety and effectiveness.

Supraperiosteal Layer Injection: Employing thick needles for high-viscosity filler injections directly onto the bone in the temporal region can be hazardous, as the absence of a distinct separation between muscle and bone in this area might lead to inadvertent muscle infiltration.

Subcutaneous Layer Injection: While some practitioners opt for injections into the subcutaneous fat due to concerns over damaging deeper structures, it is critical to note that the sentinel vein traverses this layer, presenting risks of venous injury and subsequent bleeding [40].

## 12. General Recommendations

Given the intricacies and potential hazards associated with facial fillers, particularly in anatomically sensitive zones:

Training and Knowledge: Practitioners must possess comprehensive training in facial anatomy and the distinct properties of various fillers.

Careful Technique: Utilizing gentle injection methods while being cognizant of anatomical landmarks can significantly mitigate complications.

Patient Awareness: Informing patients about possible risks and symptoms of complications is vital to ensure they seek immediate medical care if adverse effects arise.

Adhering to these guidelines helps practitioners minimize risks associated with facial fillers, promoting safer patient outcomes [42,53].

## 13. Methods to Prevent Vascular Complications

Vascular complications from filler injections can result in severe outcomes, necessitating the highest level of caution during facial treatments. To diminish the likelihood of such complications, it is imperative to possess a profound understanding of the anatomical structures and to adopt safe and measured treatment practices.

## 14. Vascular Structures

Unlike surgical interventions where visibility of internal structures is possible, filler injections are typically performed blindly, heightening the potential for inadvertent damage. Vascular structures, embedded within the subcutaneous layers, are particularly prone to complications due to their three-dimensional trajectory which includes depth and horizontal spread. The facial arterial system is mainly derived from the external carotid artery (ECA) and the internal carotid artery (ICA), with blindness as a critical risk when injections inadvertently reach the central retinal artery—a branch of the ICA. Recognizing the interconnected nature of these arterial systems is crucial for avoiding injections that might propagate to the central retinal artery [11].

To minimize vascular risks, injections should preferably be administered into areas with minimal arterial presence, such as the supraperiosteal layer known for its reduced vascularity. Additionally, directions of injections should be strategically chosen to lessen potential vessel damage. Caution is particularly warranted in regions with previous surgical or traumatic scarring, where altered vascular integrity could predispose to heightened risks.

### Appropriate Material and Tool Selection

For novices, the use of hyaluronic acid (HA) fillers is recommended because they can be dissolved with hyaluronidase if inadvertently injected into a vessel. The selection of filler viscosity should align with the ease of injection and the need for precision in molding; excessively viscous fillers may necessitate undue force, complicating the injection process for beginners [48,54,55,56,57].

The syringe’s barrel diameter also influences the injection force, with smaller diameters offering better control for inexperienced practitioners. The decision between using a needle or a cannula should be made judiciously: while cannulas, with their blunt ends, are generally safer in terms of vascular penetration, they must be maneuvered with care to avoid misplacement. Needles, though offering more precise placement due to their sharpness, pose a greater risk of vessel penetration [52,58].

Effective prevention of vascular complications ultimately hinges on a thoughtful selection of tools and materials, tailored to the practitioner’s level of expertise and the specific demands of the injection site and layer. Continuous training and experience are essential for optimizing safety and efficacy in filler injections.

## 15. Needle vs. Cannula

Previously, it was commonly believed that cannulas posed a lower risk of vascular damage during filler injections than needles. However, emerging studies suggest that the incidence of complications with cannulas might be comparable to or exceed those with needles, sparking debate within the medical community and highlighting the need for ongoing research [52].

Needles and cannulas have distinct structural differences: needles are sharp and rigid, providing precise delivery, whereas cannulas are blunter and more flexible, theoretically reducing the likelihood of penetrating a vessel. Yet, this flexibility can lead to inaccurate placement, potentially driving the cannula into vascular-rich layers contrary to intended superficial placements. Additionally, the perceived safety of cannulas might encourage more aggressive use, potentially increasing the risk of complications [5].

Despite these concerns, cannulas remain the recommended choice for beginners due to their blunt ends which inherently reduce the risk of puncturing vessels. Experienced practitioners should judiciously choose between needles and cannulas based on the specific requirements of the procedure and their familiarity with each tool.

### 15.1. Diameter of Needle and Cannula

The probability of a needle or cannula entering a vessel inversely correlates with its thickness relative to the vessel size. For instance, finer cannulas (27–30 G) are more likely to penetrate small vessels (around 1 mm in diameter) than thicker ones (23 G).

In practice, thicker cannulas (21 G to 23 G) are recommended for deeper injections near muscular layers harboring larger vessels, while thinner cannulas (25 G to 27 G) are preferable for superficial injections close to smaller vessels. Similarly, needles ranging from 23–25 G are typically used for deeper injections, with finer 30 G needles reserved for superficial layers [12].

It is crucial to understand that while smaller cannulas are flexible and less likely to penetrate vessels due to their bluntness, their use does not eliminate the risk of piercing vessel walls. Moreover, the assumption that thicker cannulas automatically provide safer injections is flawed, as some facial vessels are large enough to accommodate a 23 G cannula [1].

The theory that smaller diameters increase injection pressure, potentially causing fillers to move rapidly through vessels, lacks robust empirical support. Instead, the concern in ocular complications is more about the linear movement of filler particles within a vessel leading to blockages near the eye or optic nerve, necessitating further research to clarify the impact of tool diameter and injection pressure on such risks [8].

### 15.2. Aspiration Test

While the aspiration test prior to filler injection is widely practiced, its definitive effectiveness remains unproven. Nevertheless, there are several considerations that can improve the reliability and efficiency of this method.

Firstly, the gauge of the needle or cannula is critical; larger diameters improve the likelihood of successful blood aspiration due to the easier establishment of negative pressure, while smaller gauges may result in false negatives due to inadequate negative pressure, preventing blood from being aspirated even if the needle or cannula is within a vessel [13,14,18,23,26,29,33,42,59,60,61,62,63,64].

Secondly, before conducting the test, ensure that the filler material is primed within the needle or cannula by advancing the plunger until the filler is slightly visible at the tip. This preparation helps prevent the filler from occluding the lumen, which can hinder blood aspiration. This is particularly important with high-viscosity fillers and smaller diameters, where the risk of occlusion and thus false negatives increases [28,32,51,56,57,58,59,60,61].

Thirdly, it is possible for the vessel wall to seal against the needle or cannula tip during aspiration, especially if the vessel is partially collapsed or the needle/cannula damages the vessel during insertion. This scenario can prevent blood from entering the needle or cannula, resulting in a false negative.

Fourthly, external bleeding from a ruptured vessel during needle insertion can be absorbed by the needle or cannula, potentially leading to a false positive result, mistakenly indicating that the needle or cannula is inside a vessel when it is not.

Fifthly, the characteristics of the filler influence the test’s outcomes. Fillers with lower viscosity and smaller particles generally allow blood to be aspirated more effectively, whereas high-viscosity fillers may show air bubbles initially under negative pressure, delaying the appearance of blood and requiring sustained suction to confirm vessel penetration.

Lastly, movement of the needle or cannula during the test can alter their position, potentially leading to negative results and unintended vessel damage. This risk is exacerbated when using high-viscosity fillers, necessitating careful, steady application of negative pressure and minimal movement during the test.

Despite these complexities, many practitioners continue to perform aspiration tests, occasionally detecting blood reflux, suggesting some utility in the procedure. Research indicates that larger, shorter needles more effectively facilitate aspiration compared to cannulas [5,52].

The aspiration test remains a valuable component of safe injection practices, particularly in vascular-rich areas. Optimal results require careful consideration of the tool gauge, filler viscosity, and technique stability. Further research and in vitro studies comparing different types of fillers and their interaction with blood during aspiration tests are ongoing, providing deeper insights into the mechanics and reliability of this precautionary measure.

**Figure 6:**
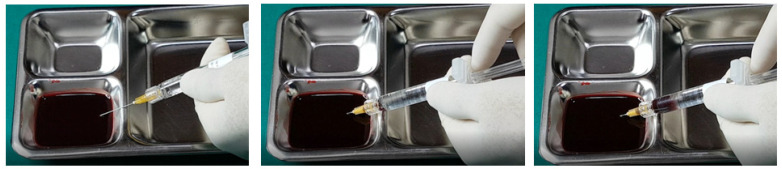
Aspiration test for highly elastic biphasic HA filler using needle.

**Figure 7:**
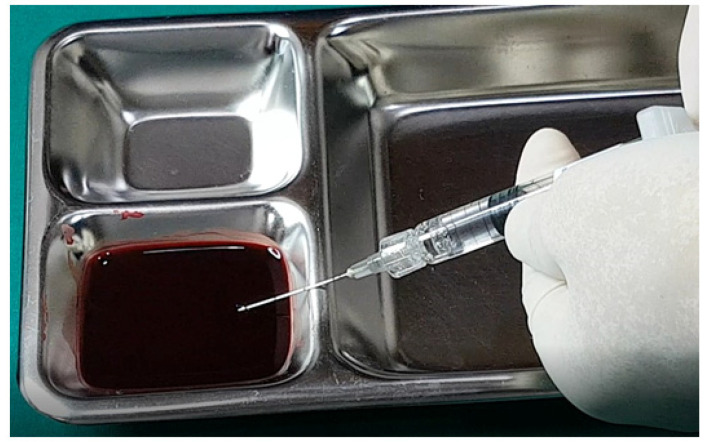
Aspiration test for highly elastic biphasic HA filler using medium-sized cannula.

**Figure 8:**
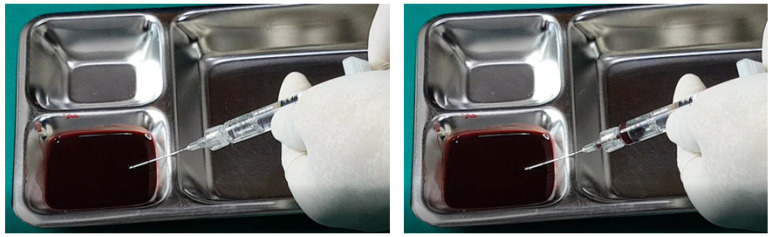
Aspiration test for highly elastic biphasic HA filler using large-sized cannula.

In examining the effectiveness of the aspiration test with high-elasticity biphasic fillers, results showed that blood was aspirable through needles of at least 25–27 G gauge, but not through a cannula of the same gauge, as depicted in Figure 6 and Figure 7, respectively. Successful blood aspiration was achievable with a 23 G cannula, as shown in Figure 8. During these tests, upon application of negative pressure, air bubbles initially filled the syringe, followed by blood that gradually occupied the space from the syringe tip to the center and wall (Figure 2, Figure 3, Figure 4, Figure 5, Figure 6, Figure 7 and Figure 8).

**Figure 9:**
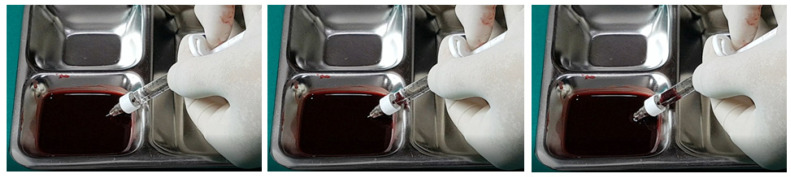
Aspiration test for medium elastic biphasic HA filler using needle.

**Figure 10:**
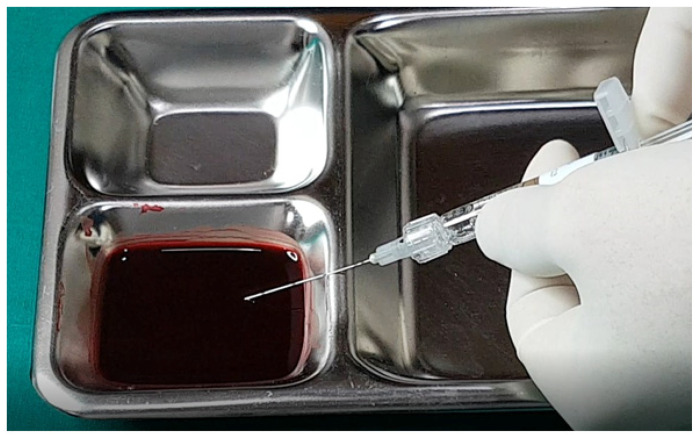
Aspiration test for medium elastic biphasic HA filler using small-sized cannula.

**Figure 11:**
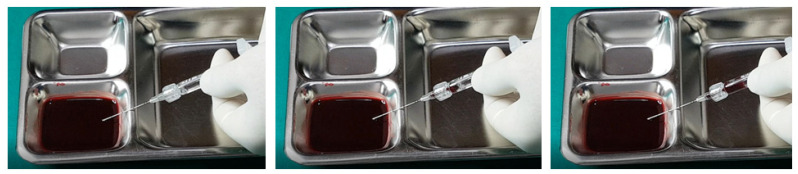
Aspiration test for medium elastic biphasic HA filler using medium-sized cannula.

Further testing with biphasic fillers of moderate elasticity demonstrated that needles sized 27–29 G could aspirate blood, as seen in Figure 9. However, a 27 G cannula failed to achieve the same result (Figure 10), whereas a 25 G cannula was successful (Figure 11). The pattern of blood and air filling the syringe was consistent with that observed in the high-elasticity biphasic fillers, detailed in Figure 9 and Figure 11.

**Figure 12:**
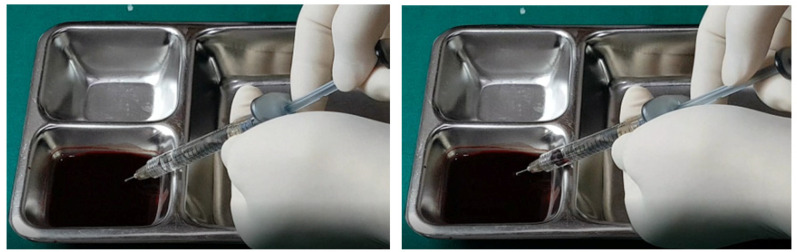
Aspiration test for monophasic HA filler using needle.

**Figure 13:**
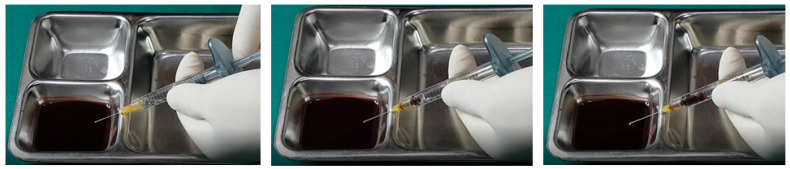
Aspiration test for monophasic HA filler using small-sized cannula.

The aspiration dynamics changed when using monophasic fillers. Needles sized 27–29 G facilitated easier blood aspiration compared to the same gauge used with biphasic fillers, as shown in Figure 12. Remarkably, a 27 G cannula, ineffective with biphasic fillers, succeeded in aspirating blood with monophasic fillers (Figure 13).

Overall, the aspiration test revealed that blood aspiration occurs more rapidly with needles than cannulas. When using needles, blood was visible at the syringe tip almost immediately after negative pressure was applied. Conversely, with cannulas, the blood appeared after a few seconds following the initial negative pressure, with air bubbles filling the syringe first (Figure 2, Figure 3, Figure 4, Figure 5, Figure 6, Figure 7, Figure 8, Figure 9, Figure 10, Figure 11, Figure 12 and Figure 13). In instances of delayed blood aspiration, it typically occurred after about 10 s of sustained negative pressure.

These findings underline the variability in aspiration efficiency based on the type of filler and the instrument used [42,52]. While needles generally provide quicker blood aspiration, cannulas require a longer waiting period, up to 14 s, to verify blood presence accurately. This difference highlights the need for practitioners to be aware of the specific characteristics of the fillers and instruments they use to ensure the safety and effectiveness of the aspiration test.

## 16. Blood Vessel-Rich Area

When conducting filler injections in areas known for a high concentration of blood vessels, it is prudent to delay immediate filler application after the placement of needles or cannulas. Instead, remove the instrument initially to check for any signs of bleeding at the entry point. If no bleeding is observed, the instrument can be carefully reinserted and the injection commenced. This precaution is equally applicable when administering local anesthesia; the absence of bleeding at the anesthesia site suggests it is safe to proceed along the same pathway for filler application.

To verify the correct placement of needles or cannulas and ensure they have not penetrated any blood vessels, conducting a suction test prior to injecting the filler is advisable. During filler injection, applying gentle pressure and utilizing a retrograde technique are recommended to enhance control and precision.

To assess the efficacy of the volumizing effect of the filler versus potential swelling from bleeding, it is beneficial to manually examine the target area both before and during the injection. This tactile assessment should be complemented by visual observation of volumetric changes. If resistance increases unexpectedly or bleeding is detected at the insertion point, pause the procedure to re-evaluate and adjust the instrument’s placement as necessary.

Regarding patient response, if the area is properly anesthetized, the patient should not experience pain during the procedure. Pain during the passage of the needle or cannula might indicate damage to vascular or neural structures, necessitating immediate reevaluation and adjustment of the treatment approach. Additionally, observe for neurological symptoms or changes in skin coloration which may indicate vascular compromise or the impact of epinephrine in the anesthetic. A pale appearance shortly after injection could signify filler embolism or vasoconstriction [2]. In such instances, monitor the area closely for about 30 min; persistent skin discoloration or worsening symptoms necessitate urgent medical intervention, whereas improvement suggests transient vasoconstriction effects.

## 17. Emerging Techniques and Technologies

The field of dermal filler treatments is evolving with new techniques and technologies designed to enhance safety and efficacy, thereby reducing the risk of vascular complications.

Advanced imaging techniques, such as High-Resolution Ultrasound (HRUS) and Infrared Imaging, provide real-time visualization of facial anatomy [65,66,67]. HRUS helps practitioners avoid blood vessels during injections, while Infrared Imaging maps out superficial and deep vascular networks before the procedure. These technologies improve precision and reduce the risk of vascular complications.

**Figure 14:**
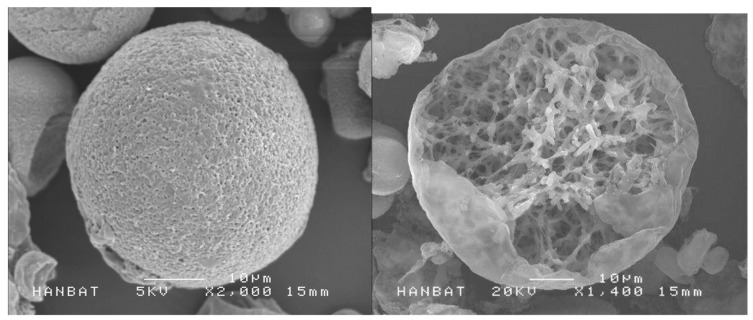
Biodegradable microspheres, like the porous-structured PDLLA (Juvelook, VAIM Inc., Seoul, Republic of Korea), gradually break down over time, minimizing long-term risks.

Innovative filler materials, like self-crossing hyaluronic acid filler and Biodegradable Microspheres, are being developed with advanced properties. Self-crossing hyaluronic acid filler has tunable properties for better integration with tissues [68]. Biodegradable Microspheres such as PDLLA (Poly-D,L Lactic Acid) with porous structure (Juvelook, VAIM, Seoul, Republic of Korea) have lesser cases of vascular incidence compared to HA-based fillers and have the benefits of dissolving over time and reducing long-term risks such as the delayed onset of non-inflammatory granulomas (Figure 14) [69,70]. Even though PLA (Poly Lactic Acid) have been used for filling purposes for decades, there are not as many vascular complications compared to fat transplantation or hyaluronic acid fillers.

Microinjection and Nanoinjection techniques use extremely fine needles for high precision in delicate areas, reducing the risk of complications and minimizing tissue trauma [71].

Technological integrations, including Augmented Reality (AR), Virtual Reality (VR), and Artificial Intelligence (AI), are transforming training and procedural planning. AR and VR provide immersive simulations for practitioners to practice complex filler injections in a risk-free environment, enhancing training and procedural accuracy [72,73]. AI analyzes patient data to predict risks and customize treatments, offering personalized treatment plans and improving procedural outcomes.

## 18. Discussion

Dermal fillers have revolutionized cosmetic procedures by offering a non-surgical solution to enhance facial aesthetics, restore volume, and smooth out wrinkles. These minimally invasive treatments provide immediate and natural-looking results, significantly boosting patient confidence and satisfaction. The versatility of dermal fillers allows for targeted enhancements, such as lip augmentation, cheekbone definition, and the reduction of under-eye hollows, making them an excellent option for personalized cosmetic improvements. Furthermore, the advancements in filler materials and techniques have increased the safety and longevity of results, ensuring that patients achieve their desired look with minimal downtime and risk. Overall, dermal fillers offer a safe, effective, and convenient method to achieve youthful and rejuvenated facial contours.

The study on vascular complications associated with dermal filler treatments highlights several critical insights and implications for clinical practice. Vascular complications are primarily attributed to two mechanisms: extravascular compression and intravascular emboli, each presenting unique challenges and risks.

Extravascular compression occurs when injected filler exerts pressure on adjacent blood vessels, leading to ischemia and potential tissue necrosis. The findings emphasize the importance of understanding facial anatomy to avoid regions where compression is more likely, such as areas with dense skin and limited space between tissues. Effective management involves prompt intervention, including massaging the area to disperse the filler and, in cases involving hyaluronic acid (HA) fillers, the use of hyaluronidase to break down the filler and reduce compressive effects. This study underscores the need for clinicians to be proficient in identifying early signs of ischemia and to act quickly to mitigate adverse outcomes.

Intravascular emboli involve the direct entry of filler into a blood vessel, leading to vessel obstruction and ischemia. The study details the rapid onset of symptoms following an embolic event and the severe consequences, including blindness and cerebral infarction. Prevention strategies include using blunt cannulas instead of sharp needles, injecting slowly with minimal pressure, and avoiding high-risk areas. For HA fillers, the use of hyaluronidase can dissolve the embolus, highlighting the importance of having this enzyme readily available in clinical settings. This study reinforces the necessity of meticulous injection techniques and thorough knowledge of vascular pathways to prevent these life-threatening complications.

The regions most susceptible to vascular complications align with major arterial pathways, such as the facial artery in the nasolabial folds and the angular and dorsal nasal arteries in the nasal region. Historical data show a higher incidence of complications in these areas due to the frequency of filler treatments. The study’s emphasis on the glabella and forehead regions, where complications have increased, calls for heightened awareness and careful injection practices in these areas.

Necrosis is a common outcome of both extravascular compression and intravascular emboli. The study categorizes necrosis into stages and outlines corresponding symptoms and management strategies. Early identification and treatment are crucial to prevent permanent damage. For instance, immediate decompression techniques, the use of vasodilators like Prostaglandin E1 (PGE1), and hyperbaric oxygen therapy are highlighted as effective interventions to improve blood circulation and promote healing.

The study describes blindness as a catastrophic complication resulting from the occlusion of the central retinal artery by filler material. The use of hyaluronidase injected into the retrobulbar space is discussed as a potential intervention to restore blood flow and mitigate vision loss. The critical need for rapid and precise application of this treatment is emphasized, underscoring the importance of training and preparedness in clinical practice.

Though less common with dermal fillers compared to fat grafting, pulmonary embolism remains a serious risk, particularly with injections near large veins in the temporal area. Prevention strategies include a thorough understanding of venous anatomy and careful injection techniques to avoid accidental intravascular injection.

The discussion on the use of needles versus cannulas reveals that while cannulas are generally considered safer due to their blunt ends, they still pose risks if not used properly. The study suggests that thicker cannulas are safer for deeper injections, while thinner cannulas and needles are suitable for superficial layers. The importance of choosing the right tool based on the specific procedure and the practitioner’s experience is highlighted.

The aspiration test is widely used to detect vessel penetration before filler injection. The study notes the limitations and potential for false negatives, especially with high-viscosity fillers and smaller gauge needles or cannulas. Despite these challenges, the aspiration test remains a valuable safety measure, particularly in vascular-rich areas.

It is advised to maintain an emergency kit with hyaluronidase and vasodilators and establish networks with experienced physicians for consultation or referral in case of complications. Patient education on recognizing symptoms of vascular occlusion and seeking prompt medical attention is also crucial. In further studies, this should be discussed.

Lastly, vascular aging involves alterations in the mechanical and structural properties of the vascular wall, resulting in the loss of arterial elasticity and decreased arterial compliance. These changes make older individuals more susceptible to vascular complications [74]. Further studies should assess the incidence of complications in relation to aging anatomy.

In conclusion, this special topic gives comprehensive guidelines and recommendations for preventing and managing vascular complications associated with dermal filler treatments. The emphasis on anatomical knowledge, careful injection techniques, early detection, and prompt intervention is essential for ensuring patient safety and optimal outcomes. Continuous education and training for practitioners, along with advancements in filler materials and injection methods, are critical for minimizing risks and improving the overall safety of cosmetic procedures.

## Figures and Tables

**Table 1:** Main symptoms of vascular complications depending on mechanism and vascular type.

Blood Vessel Type	Intravascular Emboli	Extravascular Compression
Artery	Skin and tissue necrosis Blindness Cerebral infarct	Skin and tissue necrosis
Vein	Pulmonary embolism	

**Table 2:** Basic stage, symptoms, and principles of treatment of skin and tissue necrosis.

Stage	Symptom	Treatment
Impending Necrosis (Ischemic state)	Skin color-purple of reticular pattern Pain swelling	Decompression: Hyaluronidase, Puncture and drainage, Warm massage Revascularization: PGE1 (vasodilator) IV injection, Hyperbaric O_2_, Aspirin
Skin Necrosis (Infection and wound healing state)	Pustule Eschar	Infection control: Antibiotics, Removal of pustule Dressing: Antibiotics gauze Growth factor: EGF/PDRN/PRP/stem cell
Scar Formation (Skin defect)	Erythematous scar Depressed scars	Scar treatment: Vascular laser, Fractional laser, Skin graft

**Table 3:** Comparison of Needle vs. Cannula for Filler Injections.

Instrument	Advantages	Disadvantages	Recommended Use
Needle	Precise delivery, Easier aspiration	Higher risk of vascular penetration	Deep injections in low-risk areas
Cannula	Blunt tip reduces vascular injury	Less precise, Possible inaccurate placement	Superficial injections in high-risk areas
